# Predicted Coverage and Immuno-Safety of a Recombinant C-Repeat Region Based *Streptococcus pyogenes* Vaccine Candidate

**DOI:** 10.1371/journal.pone.0156639

**Published:** 2016-06-16

**Authors:** Celia McNeilly, Samantha Cosh, Therese Vu, Jemma Nichols, Anna Henningham, Andreas Hofmann, Anne Fane, Pierre R. Smeesters, Catherine M. Rush, Louise M. Hafner, Natkuman Ketheesan, Kadaba S. Sriprakash, David J. McMillan

**Affiliations:** 1 Bacterial Pathogenesis Laboratory, QIMR Berghofer Medical Research Institute, 300 Herston Rd, Herston, QLD, 4006, Australia; 2 Inflammation and Healing Research Cluster, School of Health and Sport Sciences, University of the Sunshine Coast, Maroochydore, QLD, 4558, Australia; 3 Australian Infectious Disease Research Centre and School of Chemistry and Molecular Biosciences, The University of Queensland, St Lucia, QLD, 4072, Australia; 4 Structural Chemistry Program, Eskitis Institute for Cell and Molecular Therapies, Griffith University, Nathan, and Queensland Tropical Health Alliance, Smithfield, QLD, Australia; 5 Australian Institute of Tropical Medicine, James Cook University, Townsville, QLD, 4811, Australia; 6 Laboratoire de Génétique et Physiologie Bactérienne, Institut de Biologie et de Médecine Moléculaires, Faculté des Sciences, Université Libre de Bruxelles, Gosselies, Belgium, and Murdoch Children Research Institute, Melbourne, VIC, 3052, Australia; 7 School of Biomedical Sciences, Faculty of Health & Institute of Health and Biomedical Innovation (IHBI), Queensland University of Technology, Brisbane, QLD, 4001, Australia; Ross University School of Medicine, DOMINICA

## Abstract

The C-terminal region of the M-protein of *Streptococcus pyogenes* is a major target for vaccine development. The major feature is the C-repeat region, consisting of 35–42 amino acid repeat units that display high but not perfect identity. SV1 is a *S*. *pyogenes* vaccine candidate that incorporates five 14mer amino acid sequences (called J14_i_ variants) from differing C-repeat units in a single recombinant construct. Here we show that the J14_i_ variants chosen for inclusion in SV1 are the most common variants in a dataset of 176 unique M-proteins. Murine antibodies raised against SV1 were shown to bind to each of the J14_i_ variants present in SV1, as well as variants not present in the vaccine. Antibodies raised to the individual J14_i_ variants were also shown to bind to multiple but different combinations of J14_i_ variants, supporting the underlying rationale for the design of SV1. A Lewis Rat Model of valvulitis was then used to assess the capacity of SV1 to induce deleterious immune response associated with rheumatic heart disease. In this model, both SV1 and the M5 positive control protein were immunogenic. Neither of these antibodies were cross-reactive with cardiac myosin or collagen. Splenic T cells from SV1/CFA and SV1/alum immunized rats did not proliferate in response to cardiac myosin or collagen. Subsequent histological examination of heart tissue showed that 4 of 5 mice from the M5/CFA group had valvulitis and inflammatory cell infiltration into valvular tissue, whereas mice immunised with SV1/CFA, SV1/alum showed no sign of valvulitis. These results suggest that SV1 is a safe vaccine candidate that will elicit antibodies that recognise the vast majority of circulating GAS M-types.

## Introduction

*Streptococcus pyogenes* (group A streptococcus, GAS) is Gram-positive bacterium responsible for a wide range of diseases in humans. These include self-limiting pharyngitis, skin infections, invasive diseases and the autoimmune sequelae post-streptococcal glomerulonephritis, rheumatic fever (RF) and Rheumatic Heart Disease (RHD). The majority of these cases occur in developing nations and Indigenous communities within developed nations, where both streptococcal carriage and infection are considered to be endemic [1–4]. It has been estimated that up to half a million people die of GAS related diseases each year [5]; hundreds of millions more suffer from the less severe diseases.

**Fig 1:**
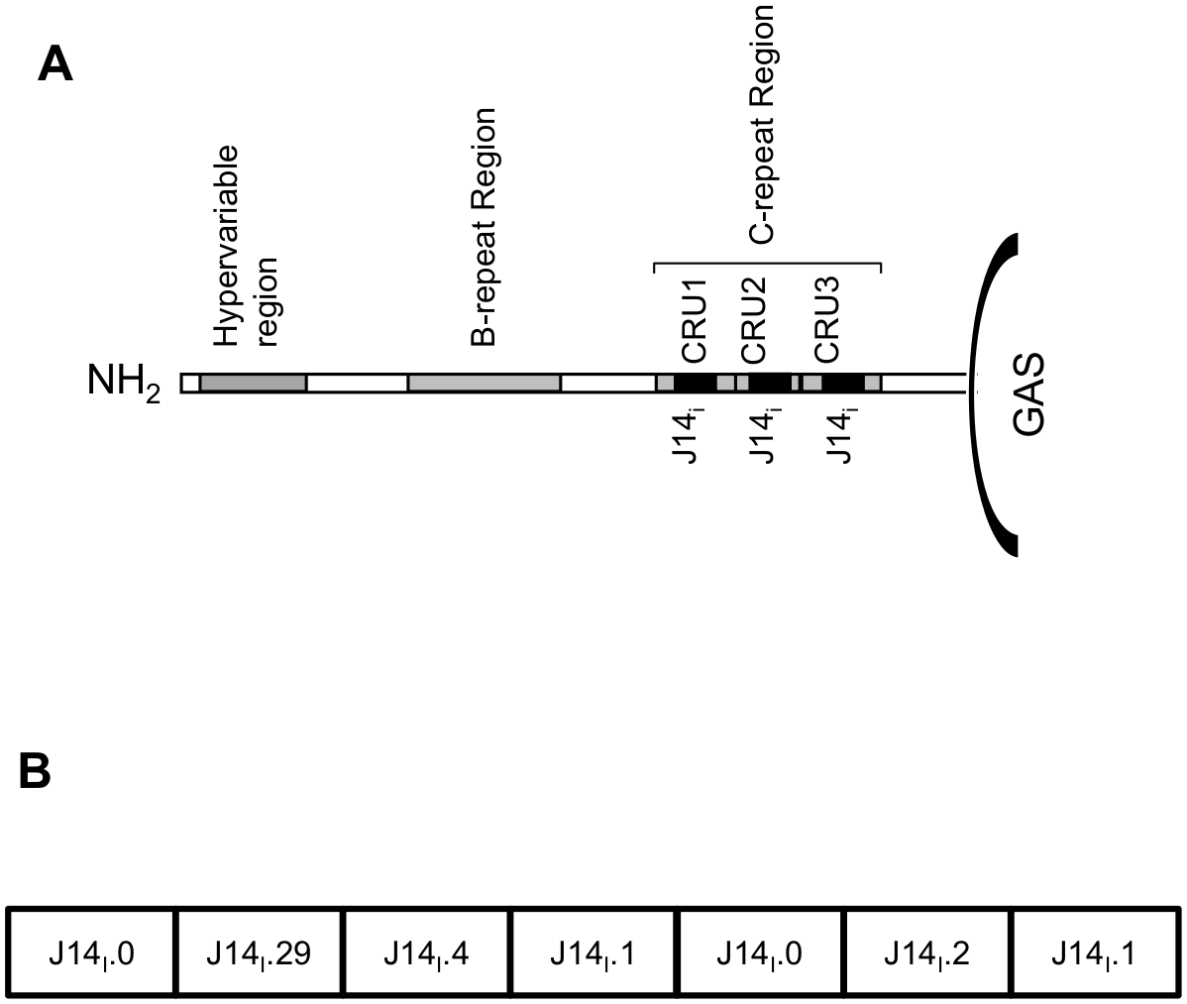
**(A) Schematic diagram of the M-protein**. The hypervariable region, B and C-repeat regions (CRR) and C-repeat units (CRUs) are depicted. The location of J14_i_ variant peptide sequences within each of the CRUs are shown as black boxes. The figure is not drawn to scale. **(B) Schematic of SV1**. The location and identity of each J14_i_ sequence is shown.

This burden of GAS disease positions the causative organism as one of the major human pathogens for which no vaccine is available. The M-protein, a major virulence determinant found on the surface of GAS, is the favored target of most vaccine development programs [6]. The major role of the M-protein is inhibition of phagocytosis through prevention of deposition of complement on the bacterial surface. The M-protein also has a secondary role as an adhesin, and has been shown to bind multiple extracellular matrix proteins [7]. Structurally the protein extends as a coiled coil dimer from the cell wall to beyond the peptidoglycan layer (Fig 1). The secondary structure of the M-protein is maintained by a repeating heptad motif that includes hydrophobic moieties at the first and fourth amino acid residues, and helix promoting amino acids at other sites [8, 9]. The amino-terminus of the M-protein is considered to be hypervariable, and used to define the more than 200 different GAS emm-types [10]. Natural and vaccine-induced antibodies to this region are bactericidal, but typically only confer emm-type specific protection [11–13]. The presence of epitopes in the B-repeat region of the protein associated with autoimmune sequelae [14] preclude its use in any vaccine candidate.

The highly conserved C-Repeat Region (CRR), found in the C-terminal half of the M-protein has been the target of several vaccine programs [15–19]. The CRR of most M-proteins contains 3 repeat units that are similar, but not identical [20, 21]. The variant sequences present in individual C-repeat units (CRU) are named on the basis of differences in an internal amino acid sequence that corresponding to the J8_i_ peptide or overlapping J14_i_ peptide sequences [21, 22]. The J14_i_ variant sequences found in these repeat units are generally conserved within an emm-type, but vary between emm-types. Currently 76 different J14_i_ types have been described. When flanked by amino acid sequence required for maintenance of alpha-helical structure, and linked to carrier molecule such as diphtheria toxoid, the prototype J8_i_ and J14_i_ peptide sequences have been shown to induce antibody responses that are bactericidal and protect mice from GAS challenge [16, 17].

Our approach to GAS vaccine development has been to identify common J14_i_ variant sequences present in different C-repeat units (CRUs) and link them into a single recombinant construct (Fig 1) [19]. SV1 contains five such sequences (J14_i_.0, J14_i_.1, J14_i_.2, J14_i_.4, J14_i_.29). Each one of the J14_i_ variant peptides present in SV1 consists of 14 amino acids. Consequently, SV1 maintains an alpha-helical structure without the need for additional flanking sequence and SV1 is also immunogenic in mice. Anti-SV1 antibodies also bind to the surface of three GAS emm-types (*emm*1, *emm*97 and *emm*65), and are bactericidal towards the two GAS emm-type (*emm*97 and *emm*65) strains tested. The aim of the current study is to assess the emm-coverage afforded by SV1 and the immuno-safety of this vaccine. We report that the five J14_i_ variants represented in SV1 are present in 97% of M-proteins [23]. Furthermore, SV1 antibodies recognize J14_i_ variants not present in the vaccine construct, potentially extending vaccine coverage to emm-types that lack representation in the SV1 vaccine construct. Finally we also demonstrate that SV1 immunization does not induce cross-reactive immune responses as determined using a Lewis Rat model of valvulitis. Together these results suggest that SV1 has significant potential as a safe and universal GAS vaccine candidate.

## Materials and Methods

### Bacteria, proteins and peptides

The GAS strains used in this study (Table 1) have all previously been described [20, 24, 25]. They represent 9 different emm-types, and one emm-negative strain. The strains also represent each of the major clusters proposed as part of the new emm-types clustering system [20]. All isolates were grown in sterile Todd Hewitt Broth supplemented with 1% neopeptone or on Columbia Blood agar containing 5% sterile horse blood. Recombinant proteins were expressed in *Escherichia coli* and purified using nickel affinity chromatography. Peptides were synthesized to 95% purity by Mimotopes (Table 2).

**Table 1:** GAS strains used in the study.

Strain	*emm*-type	*emm*-cluster	CRU1	CRU2	CRU3
JRS145	*emm*-negative				
PRS8	*emm*12	A-C4	J14_i_.16	J14_i_.2	J14_i_.0
PRS9	*emm*19	M19	J14_i_.4	J14_i_.2	J14_i_.0
PRS15	*emm*48	E6	J14_i_.36	J14_i_.1	J14_i_.1
PRS20	*emm*60	E1	J14_i_.12	J14_i_.1	J14_i_.1
PRS30	*emm*83	D4	J14_i_.2	J14_i_.2	J14_i_.0
PRS42	*emm*99	E6	J14_i_.36	J14_i_.1	J14_i_.1
PRS55	*emm*9	E3	J14_i_.29	J14_i_.1	J14_i_.1
PL1	*emm*54	D1	J14_i_.4	J14_i_.2	J14_i_.0
5448	*emm*1	A-C3	J14_i_.2	J14_i_.2	J14_i_.0
5448 AP	*emm*1	A-C3	J14_i_.2	J14_i_.2	J14_i_.0
5448Δ *hasA*	*emm*1	A-C3	J14_i_.2	J14_i_.2	J14_i_.0

**Table 2:** Peptides used in the study.

Peptide	Sequence	%TFE required for helical structure
J14.0_i_	ASREAKKQVEKALE^a^	40
J14.1_i_	ASREAKKKVEADLA	40
J14.2_i_	ASREAKKQVEKDLA	40
J14.4_i_	ASREAKKQLEAEHQ	20
J14.29_i_	ASRAAKKDLEAEHQ	>50
J14.8_i_	ASRAAKKELEAEHQ	>50
J14.12_i_	ASRAAKKELEAKHQ	40
J14.36_i_	ASRAAKKELEANHQ	40
J14.53_i_	GSRAAKKELEAKHQ	20
J14.57_i_	ASRAAKKELEAKHQ	40
J14.0_i_	KQAEDKVKASREAKKQVEKALEQLEDKVK	
J14.1_i_	KQAEDKVKASREAKKKVEADLAQLEDKVK	
J14.2_i_	KQAEDKVKASREAKKQVEKDLAQLEDKVK	
J14.4_i_	KQAEDKVKASREAKKQLEAEHQQLEDKVK	
J14.29_i_	KQAEDKVKASRAAKKDLEAEHQQLEDKVK	
J14.8_i_	KQAEDKVKASRAAKKELEAEHQQLEDKVK	
J14.12_i_	KQAEDKVKASRAAKKELEAKHQQLEDKVK	
J14.36_i_	KQAEDKVKASRAAKKELEANHQQLEDKVK	
J14.53_i_	KQAEDKVKGSRAAKKELEAKHQQLEDKVK	
J14.57_i_	KQAEDKVKASRAAKKELEAKHQAQLEDKVK	

^a^ The underlined section of each 14mer peptide indicates predicted α-helical structure, as determined by PSIPRED.

### Murine immunization

Female six-week old BALB/c mice (Animal Resources Centre, Perth, Australia) were subcutaneously immunized with 25μg of antigen admixed in a 1:1 ratio with alum delivered in a final 50μ volume. Secondary, tertiary and quaternary boosts consisting of 10μg protein emulsified 1:1 with aluminium hydroxide were delivered on days 21 and 28 and 35. Blood was collected prior to each immunization and 7 days after the final immunization. Sera from the final bleed was used in all assays reported here. All murine work was approved by the QIMR Berghofer Animal Ethics Research Committee (A0311-641).

### Secondary structure

The secondary structure of 14mer peptides in solution was investigated by circular dichromic spectra acquired with a JASCO J-715 CD Spectropolarimeter at 20°C. Peptide solutions were assessed at 0.02 mg/mL in 10 mM sodium phosphate, 20 mM NaCl, pH 8.0. Far UV-CD spectra were acquired at from 260 to 190 nm in 0–50% 2,2-trifluoroethanol (TFE). All spectra were corrected for buffer baseline and converted to mean residue ellipticity (θ) using the software ACDP v2.9 [26] and compared with the software SDAR v2.9 [27]. The amount of TFE required to observe alpha-helical secondary structure is based on the appearance of a peak at 208 nm. For each peptide, secondary structure was predicted based on its amino acid sequence using the software PSIPRED [28].

### ELISA

The wells of PVC micro-titer plates were coated with 100μl of a solution containing 500ng/ml peptide or protein diluted in 0.05M sodium carbonate coating buffer (pH 9.6). After incubation, the wells were blocked with 5% skim milk in PBS containing 0.05% Tween20 (PBS-T) and incubated at 37°C for 90 minutes, or overnight at 4°C. Two fold serial dilutions of sera were made and incubated at 37°C for 90 minutes. The sera were decanted and plate washed with PBS-T prior to addition of HRP conjugated goat anti-mouse secondary antibody (AbD Serotec) diluted 1:3000 in 0.5% skim milk/PBS-T. After incubating at 37°C for 90 minutes the plates were washed, *o*-Phenylenediamine Dihydrochloride was added to the wells, and incubated again at room temperature. The absorbance (450nm) was then measured. Antibody titers were defined as the reciprocal of the highest dilution of samples that yielded an optical density at 450nm of more than 3 standard deviations above the mean optical density of pre-immune sera. Statistically significant differences in ELISA titers were assessed using ANOVA and Kruskal Wallace non-parametric tests.

### Immunofluorescent Microscopy

Immunofluorescent microscopy was carried out essentially as previously described [29]. Briefly, bacteria were grown overnight, washed, and fixed to a polylysine coated slide (ProSciTech) with 3% paraformaldehyde. The fixed slides were then blocked with 5% skim milk/PBS-T. To prevent non-specific Fc-IgG binding by streptococcal surface proteins the slides were exposed to a second blocking step using non-specific human polyclonal IgG (AbD Serotec) was undertaken. The slides then were incubated with pooled murine antisera (1:200), washed, and incubated with secondary goat anti-mouse-IgG-FITC labelled antibody (Invitrogen, USA) diluted 1:200 in 0.5% skim milk/PBS-T. The slides were mounted in Prolong Gold (Life Technologies) and viewed with a 100x objective lens using a 473nm laser with absorbance of 500-650nm on an Olympus Fluoview FV1200 Confocal Laser Scanning microscope.

### Western Blotting

Purified recombinant M-protein, porcine cardiac myosin (Sigma-Aldrich) and skeletal muscle tropomyosin (Sigma-Aldrich) were electrophoresed on 12% or 4–15% gradient SDS-PAGE gels, transferred to Hybond C nitrocellulose membranes (Amersham, USA), and blocked with 5% skim milk/PBS-T. The membranes were then incubated with murine anti-SV1 sera diluted 1:2000 in 5% skim milk/PBS-T, washed, and secondary goat anti-mouse IgG-HRP diluted 1:10,000 in PBS-T added. Following 60 min incubation, the membrane was washed, covered with chemiluminescent substrate (ECL Western Blotting Detection Reagent, Amersham) following manufactures instructions and exposed to standard X-ray film.

### Immonosafety of SV1

The immuno-safety of SV1 was evaluated in a Lewis rat model of autoimmune valvulitis [30, 31]. Lewis rats (LEW/sSN; Albino: a,h,c: RT1) were purchased from the Animal Resources Centre (Canning Vale, Western Australia) and bred by sibling mating in Small Animal Breeding Facility at James Cook University (Townsville, Australia). For all immunisations rats were initially anaesthetised using 5% Isoflurane (Provet, Queensland, Australia) and 1–2 L/min O2. The Isoflurane level were subsequently maintained at 2% during immunization procedures. Following immunisation a local anaesthetic cream containing 25mg/g lignocaine and 25 mg/g prilocaine was applied to the injection site. Eight to twelve week-old female Lewis rats were subcutaneously immunized on day 0 in the hock of the hind left foot with 0.5 mg of SV1 or recombinant M5-protein emulsified with Complete Freund's Adjuvant (CFA), or intramuscularly with the same proteins admixed in 2% alum. Negative control rats were immunized with PBS in adjuvant. On days 1 and 3, the rats were intraperitoneally injected with 0.3μg *Bordetella pertussis* toxin (Sigma, Australia). Antigen boosts consisting of 0.5 mg of protein emulsified 1:1 with incomplete Freund's adjuvant, or admixed 2% alum were subcutaneously delivered on day 7. All animals were euthanased on day 21 by CO2 asphyxiation in a lethal chamber, followed by cervical dislocation. Blood, hearts and spleens were then collected.

To determine sera immunoglobulin G (IgG) reactivity, 10 μg/ml of M5, SV1, porcine cardiac myosin of collagen-1 (Sigma-Aldrich, St. Louis, MO) were coated onto Nunc Maxisorp plates (Roskilde, Denmark) overnight and test sera added with controls. A secondary goat anti-rat IgG (Abd Serotec) was used before OD was measured at 405nm in an ELISA plate reader. Mononuclear cells were aseptically isolated from spleens and T cell proliferation assays performed. Mononuclear cells from each group of rats were cultured in triplicate at 10^5^ cells/well in RPMI-1640 culture medium with supplement. Cells were stimulated by the addition of 10 μg/ml SV1, M5, porcine cardiac myosin or collagen type 1 or 5 μg/ml concanavalin A (ConA). The cells were pulsed with 0.25 μCi 3H-thymidine (GEHealthcare, Australia), harvested onto filter mats, and counts per minute (CPM) determined using a Microlux beta scintillation counter [25, 26]. Proliferative responses are reported as the change in CPM between stimulated and unstimulated cells. Histological studies on cardiac tissue were carried out on rat hearts fixed in neutral buffered formalin and embedded in paraffin. Sections were stained with hematoxylin and eosin (H&E) and examined under a light microscope for evidence of valvulitis. All experiments using the rat model of valvulitis were approved by the James Cook University Ethics Committee (A1688).

### FACS analysis

FACS was used to assess the binding of anti-sera to the surface of three GAS isolates that differed in their capsule expression levels [19]. GAS 5448 is an *emm*1 clinical isolate associated with invasive disease outbreaks [32]. GAS 5448AP is a hypervirulent derivative of GAS 5448 that, due a CovRS mutation, overexpresses capsule [33, 34]. GAS Δ*hasA* possesses a mutation in the capsular encoding *hasA* genes, resulting in abrogation of capsule expression. Bacteria were grown to mid log-phase, washed in PBS, and then incubated with human IgG in 0.3% BSA/PBS/Tween to block non-specific binding. The cells were sequentially incubated with primary antibody, followed by secondary goat anti-mouse FITC conjugated IgG, washed and fixed in 4% paraformaldehyde. Fluorescence was measured using a FACSort flow cytometer, and mean fluorescence intensity (MFI) determined. Statistical significant differences in MFI between PBS and experimental groups was subsequently assessed using by t-test.

## Results

### J14_i_-variants present in SV1 are common in 97% of 173 emm-types

The selection of the J14_i_ variants present in SV1 was originally made after the examination of genes representing 77 different emm-types that were present in the Genbank database [19]. A subsequent study of emm-gene sequences of greater than 1000 globally distributed GAS isolates provided the opportunity to expand the analysis of the coverage of J14_i_ variants represented in SV1 to a larger dataset that include 173 unique emm-genes [23]. As the earlier study showed that the C-terminal amino acid sequences to be highly conserved within an emm-type, and to avoid bias due to over-representation, individual representatives of each emm-type were selected for the current analysis. Of the 173 unique emm-genes, all but five (emm134, emm174, emm137, emm205 and emm211) contained at least one of the J14_i_ variants represent in SV1 (S1 Table). Sixty (35%) of these putative M-proteins possessed one variant, 90 (52%) possessed two variants, and 18 (10%) possessed three of these variants. J14_i_.0 and J14_i_.1 were the two most common variants, present in 43% and 51% of emm-types respectively. J14_i_.2, J14_i_.4 and J14_i_.29 were present in 41%, 21% and 12% of the emm-types respectively. These latter three variants were the most prevalent J14_i_ variants in CRU1, the CRU most distant from the bacterial surface (Table 3). J14_i_.0 was only found in the CRU1. Finally, 512 individual CRUs were present within the 173 M-proteins analyzed. Of these, 422 possessed a J14_i_ variant present in SV1. Together these results demonstrate that J14_i_ sequences present in SV1 are present in 97% of the emm-types examined in this study, and account for the majority of all J14_i_ sequences.

**Table 3:** Distribution of J14_i_ variants in CRUs.

	% distribution of J14_i_-variants			
	CRR1	CRR2	CRR3	All
**J14**_**i**_**.0**	0.0	0.0	45.47	44.6
**J14**_**i**_**.1**	4.6	44.8	49.1	50.8
**J14**_**i**_**.2**	26.3	36.6	0.6	41.7
**J14**_**i**_**.4**	20.4	8.7	0.0	21.1
**J14.29**_**i**_	13.2	1.16	0.0	12.0

### SV1 antibodies recognize multiple J14_i_ variants

**Fig 2:**
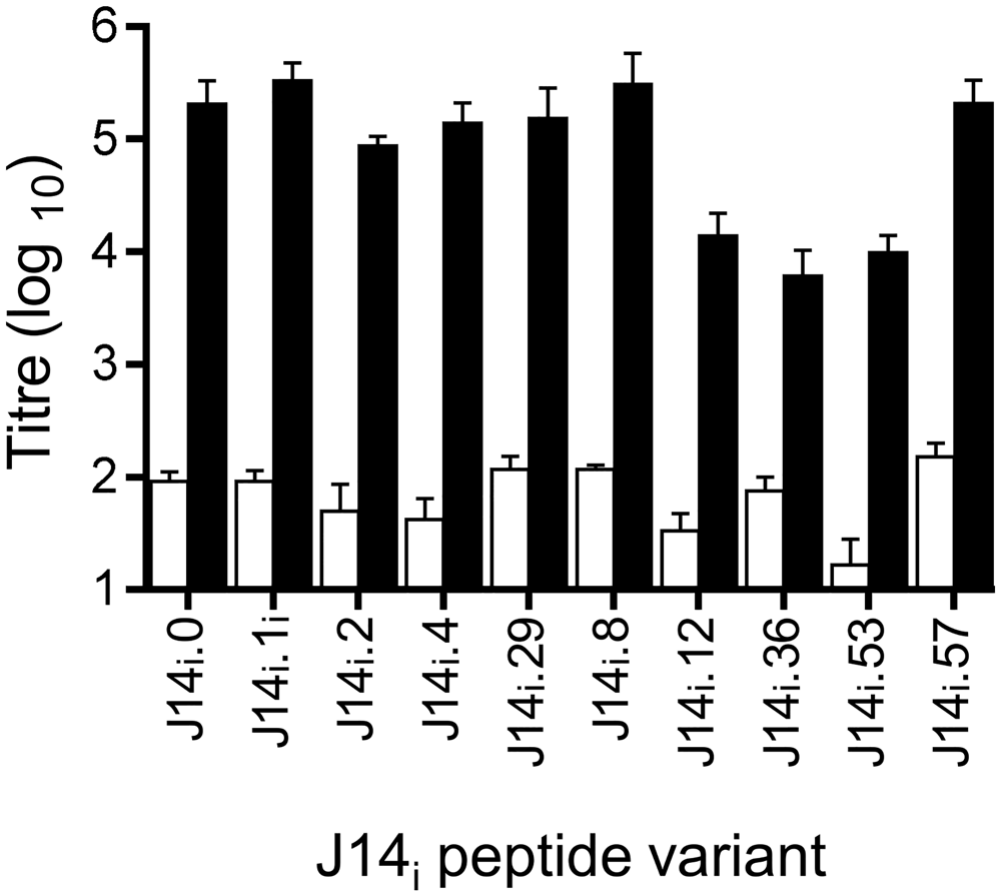
Peptide specific serum IgG responses of mice immunized with SV1. Data is presented as the mean ± standard deviation of ten mice per group. Anti-SV1 titers were significantly greater than anti-PBS titers for all peptides (p < 0.05). Black bars represent mean titers observed using anti-SV1 antisera. Open bars represent mean titres observed using anti-PBS antisera.

To show that anti-SV1 antibodies did actually recognise and bind to the individual J14_i_ variants represented in the vaccine, sera collected after final immunisation of mice was used in ELISA against a panel of J14_i_ variant peptide sequences (Fig 2). The panel included the variants present in SV1 and five additional J14_i_ sequences. These latter peptides were included to assess the capacity of SV1 antibodies to bind other J14_i_ sequences and were selected for inclusion on the basis of their ubiquity (J14_i_.8, J14_i_.12, J14_i_.8, J14_i_.36) or because they possessed unique sequences at specific amino acid sites (J14_i_.53 and J14_i_.57). The mean titers for the five SV1 represented variants ranged from 3.3x10^5^ ± 9.6 x10^4^ to 8.7 x10^4^ ± 2.5 x10^4^. For the peptides not present in SV1, the highest titers were observed using J14_i_.8 (3.1x10^5^ ± 2.5 x10^4^) and anti-J14_i_.57 (2.1x10^5^ ± 2.5 x10^4^). There were no significant differences between the mean titers observed for these two peptides, and those observed with any of the peptides presents in SV1 as determined using by ANOVA and Kruskal Wallace non-parametric test. Titers against the remaining three other peptides (J14_i_.12, J14_i_.36 J14_i_.53) were lower, but still significantly higher than titers observed for PBS sera negative control sera. Together these results demonstrate anti-SV1 antibodies are capable of recognizing multiple J14_i_ variants, increasing targets for SV1 antibodies on the bacterial surface, and potentially increasing the emm-type coverage afforded by SV1.

To determine which variants present of SV1 contribute to cross-recognition, antisera were raised against J14_i_ peptides. As individual J14_i_ peptides are poorly immunogenic, the J14_i_ peptides were resynthesized with GCN4 flanking amino acid sequences, designed to maintain the alpha-helical conformation of the parental M-protein, and conjugated to diphtheria toxoid prior to immunization of mice. Sera from individual mice in each group were then pooled and used in ELISAs against the panel of 10 J14_i_ variant peptides. For these assays, a titer of 1000 was chosen as an arbitrary cut-off value representing low or no binding between sera and peptide (Table 4). As expected, antibody titers for individual sera were highest when used against the corresponding J14_i_ peptide sequence as capture antigen. Anti-J14_i_.0 anti-sera also bound to J14_i_.1, J14_i_.2, J14_i_.29 and J14_i_.57. Titers for anti-J14_i_.1 anti-sera were greater than 1000 against an additional two peptide sequences. Sera raised to J14_i_.2 and J14_i_.4 recognised 4 and 6 peptides respectively. Despite showing low titers against J14_i_.29, anti-J14_i_.29 anti-sera also bound to two peptides. Collectively, nine of the ten peptides examined were recognized by at least one of these peptide antisera. The differences in peptides recognized by the different anti-sera underscore the utility of incorporating multiple J14_i_ variants in a single construct.

**Table 4:** Relative cross-recognition between peptide specific antisera and J14_i_-variant peptides.

		Peptide									
		J14_i_.0	J14_i_.1	J14_i_.2	J14_i_.4	J14_i_.29	J14_i_.8	J14_i_.12	J14_i_.36	J14_i_.53	J14_i_.57
**Anti-sera**	**J14.0**	+++^a^	+	++	-	+	-	-	-	-	++
	**J14.1**	++	+++	-	-	-	-	-	-	-	+
	**J14.2**	+	++	++	-	-	-	-	-	-	++
	**J14.4**	-	+	-	+++	+	+	++	-	+	-
	**J14.29**	-	-	-	+	+	+	-	-	-	-

^a^ Titers less than 1000 (-); titers between 1000–10,000 (+); titers between 10,000–100,000 ‘++’; titers greater than 100,000 ‘+++’

**Fig 3:**
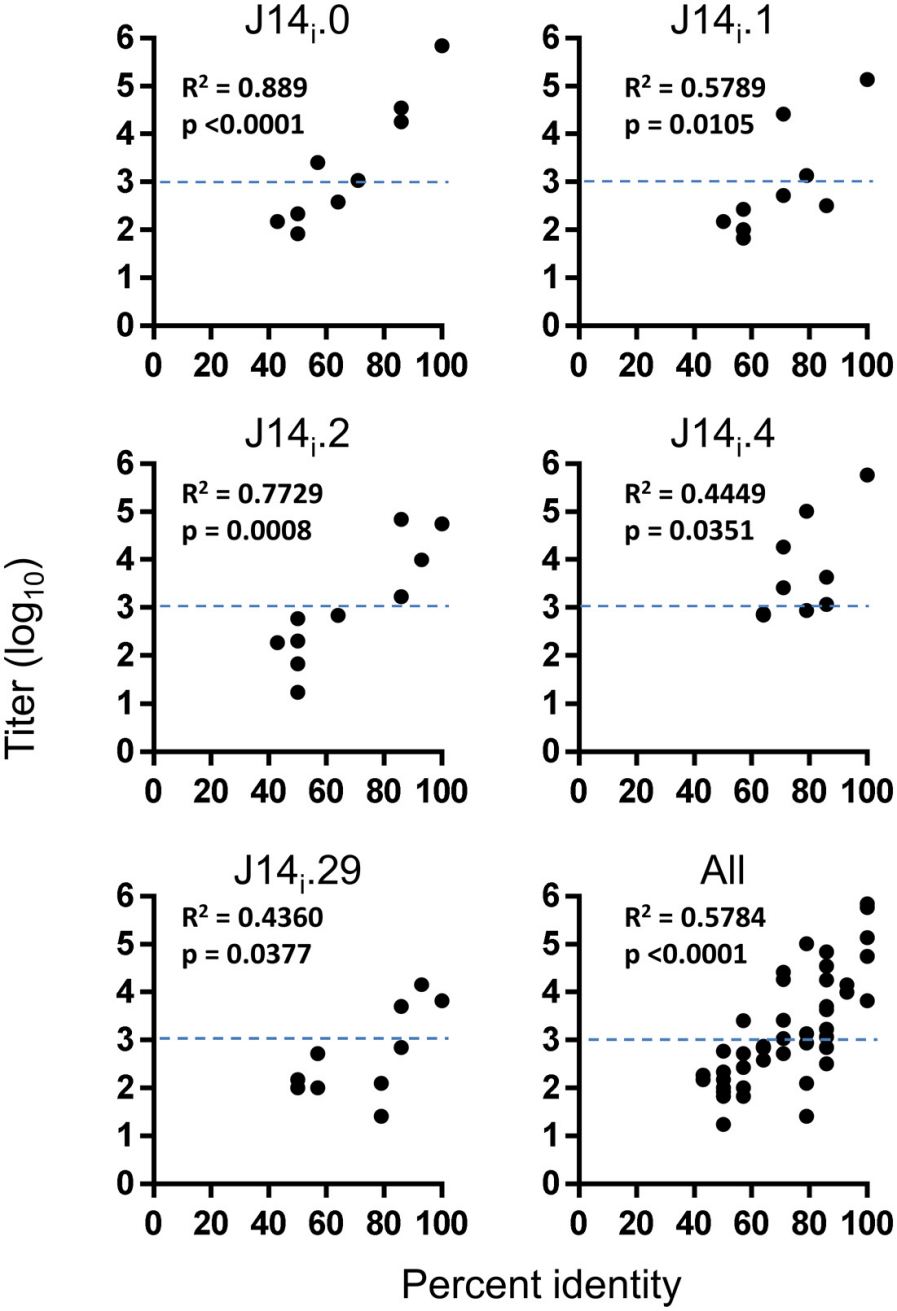
Correlation between antibody titer and peptide identity. The ELISA titer for each J14_i_ peptide antisera/peptide combination is plotted against the percent identity of the two corresponding peptides. Percent identity is plotted on the x-axis. Corresponding titers are plotted on the y-axis. Linear regression revealed that a significant correlation between these two variables exists for each antisera. The dotted horizontal line shows the cut-off used as the titre used to differentiate between low or non-significant binding, and significant binding.

To identify factors that may contribute to the cross-recognition, the percentage identity between J14_i_ peptides was determined (Table 5), and compared to the corresponding ELISA titer (Fig 3). This analysis has shown that for sera tested, there was a correlation between titer and relative identity between peptides. Using a titer of 1000 as the cut-off point as described above, cross-recognition was observed in 15 of 23 cases where the identity between J14_i_ pairs was greater than 70%, but only one instance amongst 23 pairs where the identity between sequences was less than 70%.

**Table 5:** Percent identity between J14_i_ peptides.

	J14_i_.0	J14_i_.1	J14_i_.2	J14_i_.4	J14_i_.29	J14_i_.8	J14_i_.12	J14_i_.36	J14_i_.53	J14_i_.57
**J14.0**_**i**_	100	71	86	64	57	50	50	50	43	86
**J14.1**_**i**_		100	86	71	57	57	57	57	50	79
**J14.2**_**i**_			100	64	50	50	50	50	43	93
**J14.4**_**i**_				100	86	86	79	79	71	64
**J14.29**_**i**_					100	93	86	79	79	50

### Anti-SV1 antibodies recognize multiple M-proteins

**Fig 4:**
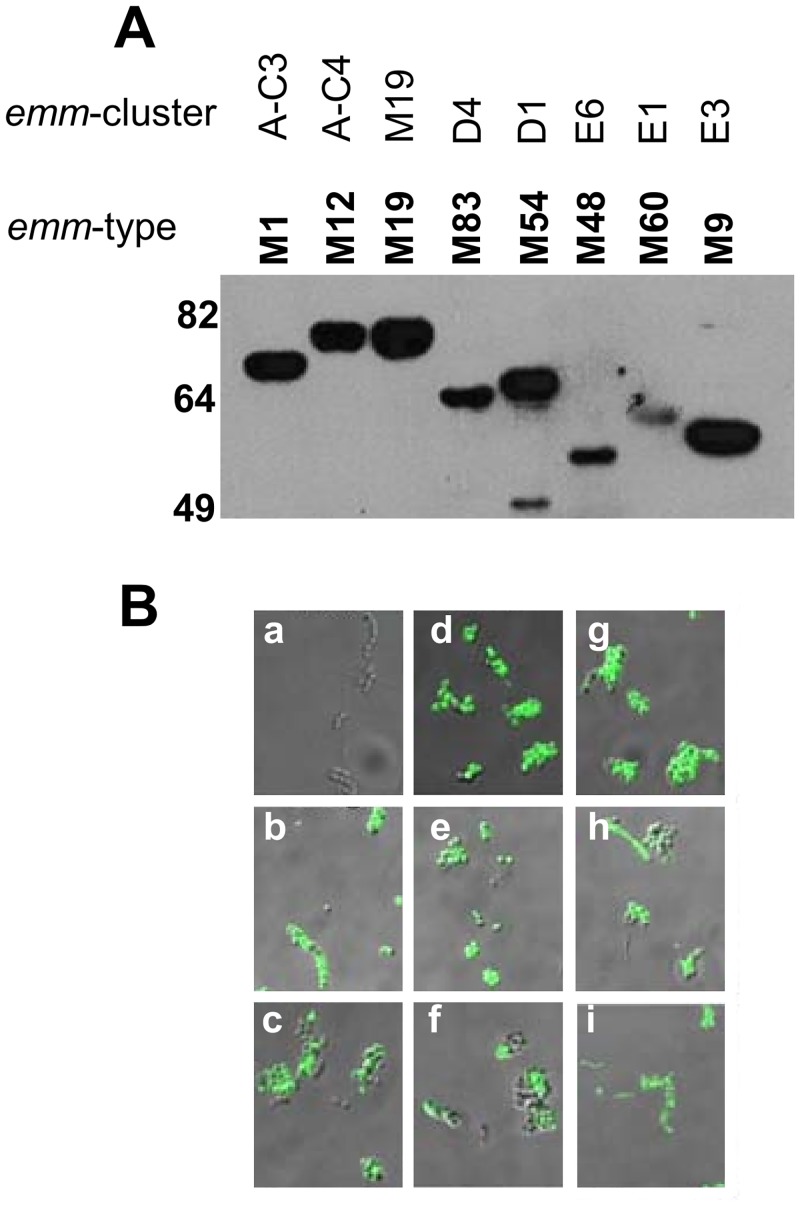
Anti-SV1 antibody binding to recombinant M-proteins and GAS surface. (**A**) M-proteins were electrophoresed in 12% SDS-PAGE gels transferred to nitrocellulose membrane and probed anti-SV1 antisera. The emm-type and *emm*-cluster assignment are shown at the top of the figure. (**B**) Immunofluorescent microscopy demonstrating that anti-SV1 antibodies bind to the surface of multiple GAS emm-types. The nine strains are (a) JRS145 (*emm*-negative), (b) 5448, (c) PRS9, (d) PL1, (e) PRS30, (f) PRS42, (g) PRS15, (h), PRS20 and (i) PRS55. Images are shown as overlays of bright field and fluorescent images. No fluorescence was observed when PBS sera was used in the same assays (data not shown).

The data above demonstrate that the design strategy for SV1 has resulted in a recombinant construct that is immunogenic and evokes antibody responses in the presence of a human approved adjuvant that theoretically should be capable of binding to the CRR of multiple M-proteins. To confirm this we subsequently conducted Western blots against eight M-proteins representing different emm-types and emm-type clusters. Binding was observed in all instances (Fig 4). We also showed that SV1 antibodies bind to the surface of eight GAS strains expressing different M-proteins, but not to the emm-negative GAS JRS145 (Fig 4).

### Immuno-safety of SV1

The presence of cross-reactive epitopes within the M-protein has been a major hurdle for all M-protein vaccine development programs. The ability to demonstrate that immune responses raised against vaccine candidates do not induce immune responses that cross-react with human proteins and tissues is an important step before vaccines can proceed to human clinical trials. Here we first used Western blots using porcine cardiac myosin and skeletal muscle tropomyosin to show that antibodies from SV1 immunised mice failed to react with these proteins (S1 Fig).

The rat autoimmune valvulitis model is the only animal model available that adequately mirrors the pathophysiological futures of RHD in humans. To provide a more robust safety assessment for SV1 as a vaccine candidate we used this model to investigate whether SV1 delivered in the presence of alum, a human approved adjuvant induced autoimmune inflammatory responses similar to that observed with RHD. For these experiments rats were immunized with PBS, recombinant M5 or SV1 in the presence of CFA. A second cohort of rats was immunized intramuscularly with the same antigens mixed with alum. Mean antibody titers to the immunizing antigen were greater than 10^5^ for SV1 and M5 groups for both antigens (S2 Fig). Significant anti-M5 titers were also observed in sera from the SV1 immunized groups. In contrast titers to SV1 and M5 were less than 100 in the PBS control groups. No cross-reactive antibodies against cardiac myosin or collagen type 1 were detected in either M5 or SV1 immunized rat sera (S2 Fig).

**Fig 5:**
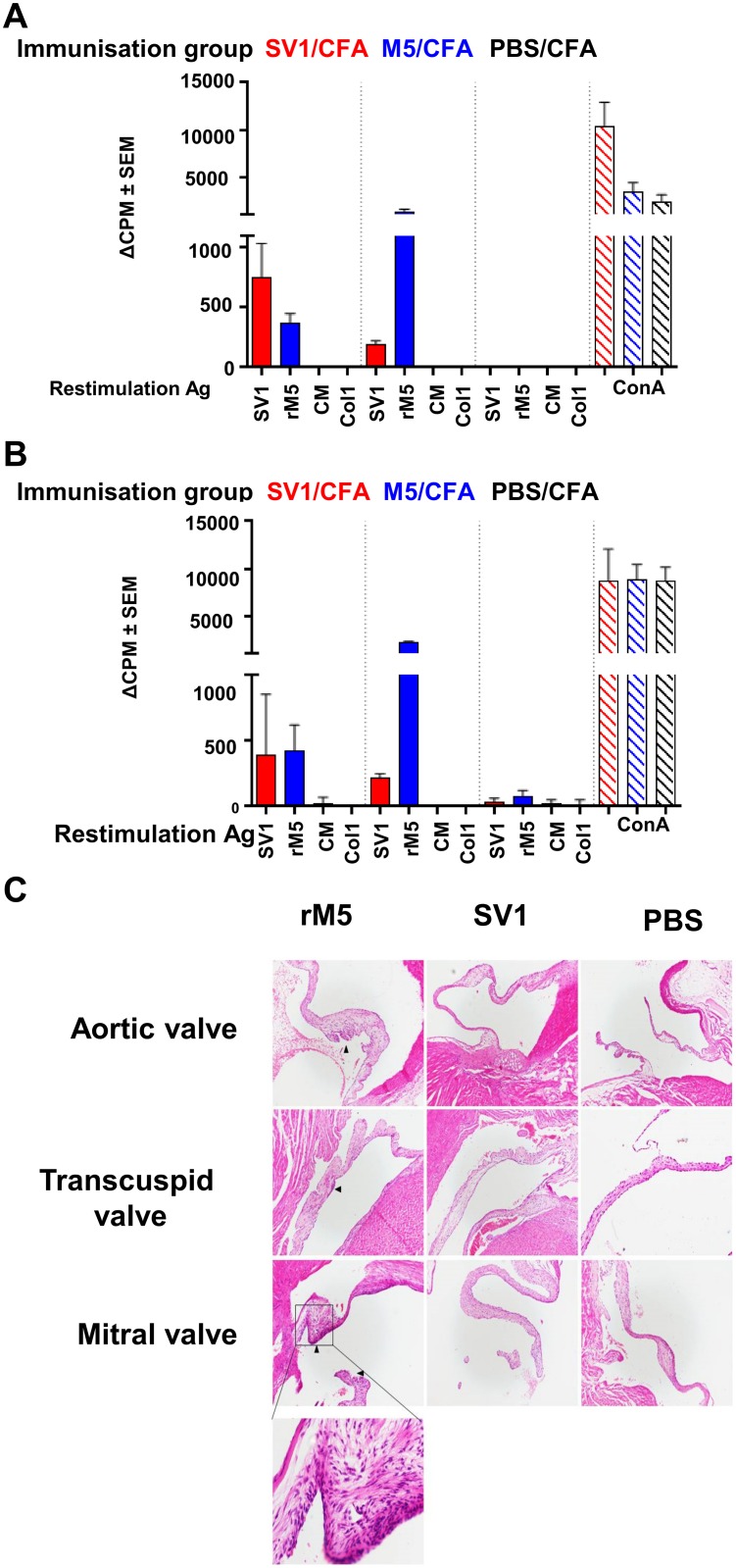
T cells from SV1 immunized rats do not cross-react with tissue antigens and do not cause inflammatory changes in cardiac tissue. (**A**) Data is presented as the change in counts per minute (ΔCPM) between stimulated and unstimulated groups (**A**) T-cells responses inSV1/CFA, M5/CFA or PBS/CFA immunized rats following restimulation with SV1, M5, cardiac myosin or collagen. (**B**) T cell responses in SV1/alum, M5/alum or PBS/alum immunized rats following restimulation with SV1, M5, cardiac myosin or collagen. (**C**) Representative histology sections of Lewis rat cardiac tissues (x100 magnification) following immunization with recombinant M5-protein, SV1 or PBS with Freund’s adjuvant. Areas of inflammation are indicated with a black arrow. A higher magnification image (x400) of an inflamed mitral valve from a rat immunized with M5 is shown. Mice that received PBS only or SV1 as antigen had no evidence of inflammatory changes in cardiac tissue.

T-cell responses to the vaccine were then assessed by measuring [^3^H] incorporation in restimulation assays (Fig 5). Splenic T cells from M5 immunized rats responded strongly to M5 restimulation irrespective of adjuvant used, weakly to SV1 restimulation, and did not respond to either cardiac myosin or collagen. Proliferative responses were greater to SV1 that M5 in the SV1/CFA immunized groups, but there were no differences in the SV1/alum immunized group. No T cell proliferative response to cardiac myosin or collagen was observed in either of the SV1 immunized groups.

Histological sections from SV1 immunized rats were compared to sections taken from M5- and PBS-immunized rats to determine whether the candidate SV1 vaccine induced inflammatory changes consistent with valvulitis or myocarditis. Four of the five rats immunized with M5/CFA had mild or moderate inflammation in the aortic and/or mitral valves. Inflammatory responses were absent in rats immunized with SV1 or PBS in Freund’s adjuvant (Fig 5). No inflammatory cell infiltrates were observed in of the groups where mice where immunized with antigen admixed with alum (S2 Fig).

### Overexpression of capsule blocks antibody binding

**Fig 6:**
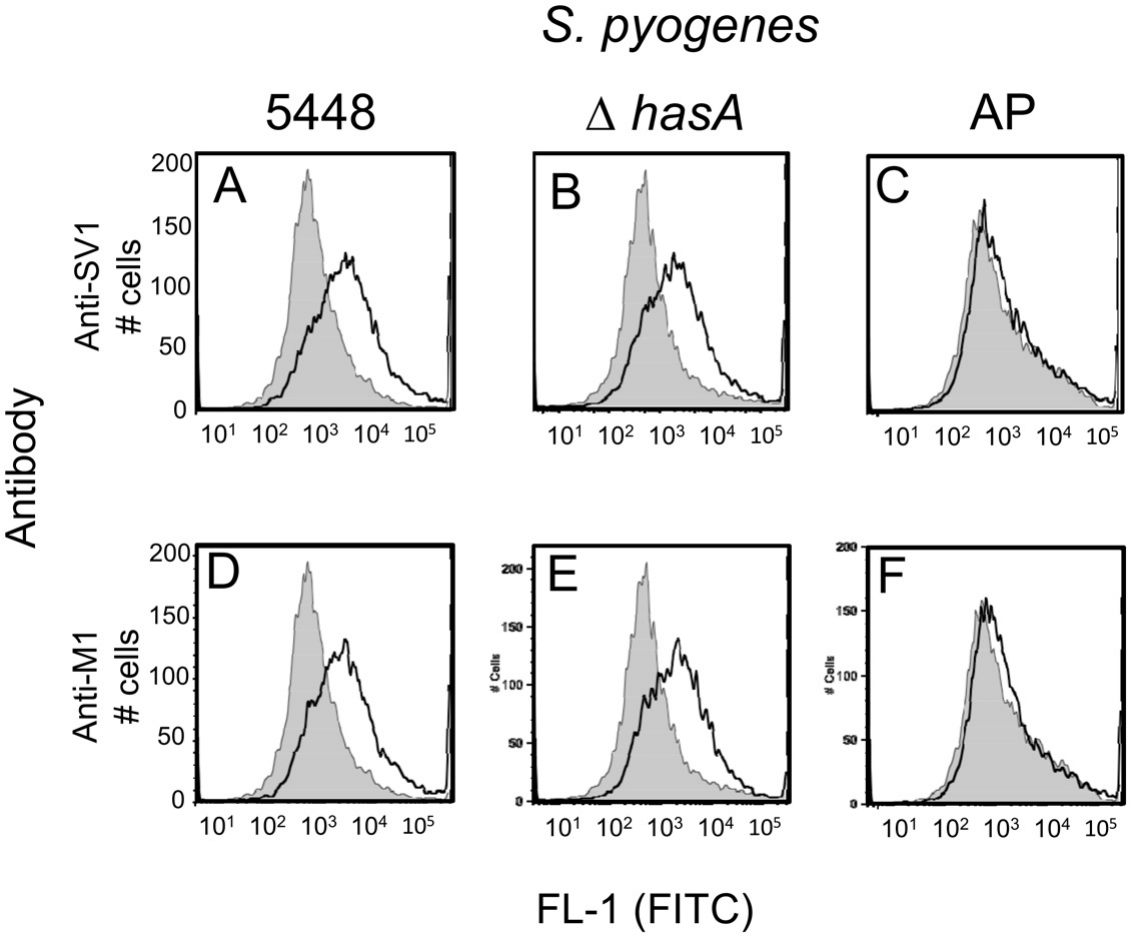
Overexpression of capsule reduces antibody binding to the surface of GAS. Representative histograms showing the binding of anti-SV1 antibodies to (A) the invasive M1 GAS strain 5448, (B) the acapsular Δ*hasA* and (C) the capsule overexpressing 5448 AP. Binding and anti-M1 antibodies to the same strains is shown in panels D-F respectively. In all images the grey shaded histogram represents binding of sera from mice immunised with PBS to GAS cells and open black lined histograms represent anti-SV1 or anti-M1-protein antibody binding to GAS strains.

Capsule is a major GAS virulence factor that, like the M-protein, also inhibits opsonophagocytosis. Total capsule expression levels differ between isolates [35, 36], are up-regulated in different host niches [37] and have been reported to block antibody binding to GRAB, another bacterial protein located on the surface of GAS. Although we saw binding to the surface of GAS in all immunofluorescent assays, it is possible that overexpression of capsule may also block SV1 antibody binding. To test this hypothesis we used a FACS based assay to compare antibody binding to wild-type GAS 5448, a derivative that hyper-expresses capsule [33] and an acapsular GAS isolate (Fig 6). In contrast to PBS sera, an increase in fluorescence was observed using anti-SV1 and anti-M1 sera against both the wild-type 5448 strain and Δ*hasA* acapsular mutant. However no significant difference in fluorescence was observed between the anti-PBS and anti-SV1 and anti-M1 sera when the hyper-encapsulated GAS 5448 AP and used in the same assay (*p* = 0.157).

## Discussion

The greatest mortality associated with GAS infection occurs due to invasive disease and the autoimmune sequelae RF and RHD [1]. The burden of these diseases is greatest in developing nations and Indigenous communities within developed nations. Epidemiological studies in these populations show that multiple *emm*-types are often circulating, and there is also no association between *emm*-type and disease outcome [21]. Here we have shown that 168 of the 173 M-proteins examined possess a least one of the J14_i_ variants present in SV1. The number of emm-types examined represents a significant proportion of all known emm-types. The five M-proteins (emm134, emm174, emm137, emm205 and emm211) possessing variants not represented in SV1 all had atypical C-repeat regions. Four of these proteins only possessed two repeat units in their CRR, with a J14_i_.12:J14_i_.40 structure. The fifth only had a single CRU harboring J14_i_.12. In the dataset analyzed here J14_i_.12 was the seventh most common J14_i_ variant, present in thirteen different *emm*-types. As we showed that anti-SV1 antibodies bound to J14_i_.12, it suggests that these emm-types would all be recognized by SV1 antisera post-vaccination. The epidemiological and cross-recognition data together suggest that in the absence of high levels of capsule on the bacterial surface, SV1 antibodies will be able to bind the surface of the majority, if not all circulating GAS emm-types.

The data reported here may also help to shed light on the coverage offered by other C-terminal GAS vaccines. Our data show that J14_i_.0 is present in 43% of emm-types. However J14_i_.0 antibodies were also shown to recognize J14_i_.1, the most abundant J14_i_ variant, and J14_i_.2, the third most common J14_i_ variant. The J8 peptide vaccine candidate includes 12 amino acids that are identical to 12 of the amino acids present in J14_i_.0. This candidate and the corresponding J14_i_ sequence have both been shown to induce protective immune responses when conjugated to diphtheria toxoid or other molecules and provided the original rationale for targeting these sequences [16, 17]. Based on this analysis here we would predict J8-DT to also provide extended M-type coverage. The StreptInCor vaccine candidate developed by Guilherme et al incorporates a 55 amino acid construct from the C-terminal region from the M5 protein and includes both human B and T cell epitopes [15, 38]. StreptInCor contains the complete sequence of the relatively rare J14_i_.6 sequence, and 12 amino acids at the C-terminus that are 100% identical to multiple J14_i_ variants. As we did not include J14_i_.6 in our analysis here, we cannot conclusively demonstrate that this sequence will induce antibodies that recognize multiple J14_i_ variants. However given the extended length of the sequence, we believe that there is a strong possibility that it will. Antibodies to StreptInCore have recently been reported to be bactericidal towards five GAS strains [39].

It is imperative that M protein based vaccine candidates do not to induce a deleterious autoimmune response following immunization. The rat model of valvulitis is a robust model that has allowed investigators to determine the development of inflammatory, immunopathological and functional changes in the rat heart that are akin to changes occurring in RHD in human [26]. Following exposure to M5 it has been shown that rat heart tissue including heart valves and the myocardium are infiltrated by inflammatory cells that include both macrophages and lymphocytes [25]. Here we tested SV1, when delivered with a human approved adjuvant, for its potential to cause autoimmune inflammatory changes that can be detected using this model. Our observations unequivocally demonstrated that SV1 does not cause any cardiac abnormalities such as those observed following M5 immunization. Therefore we conclude that SV1 is a safe vaccine candidate that does not have the capacity to induce an autoimmune response.

Finally our results also suggest that increased capsule production may influence the capacity of anti-SV1 antibodies to access antibody binding sites in the C-repeat region, a finding which may be also relevant to all GAS vaccine candidates targeting this and other surface antigens [40]. Historically, highly encapsulated strains of GAS are generally considered to be more virulent than isolates with expression lesser capsule, and are associated with rheumatic heart disease and invasive disease [36, 41]. The role that deployment of a GAS vaccine will play in the epidemiology of GAS is currently unknown. However as strains that expressing high levels of capsule are already circulating, the partial or complete blocking of antibody binding to these isolate may give them increased fitness when compared to strains expressing lesser amounts of capsule [35, 36]. Overcoming these potential limitations of CRR-targeting vaccines, and indeed all vaccine targeting proteins on the bacterial surface [13, 42–44], remains a major challenge of GAS vaccine development.

## Supporting Information

S1 Fig Anti SV1 antibodies do not cross react with cardiac proteins or tissue. **(A)** Western blot of porcine cardiac myosin (lane 1), porcine tropomyosin (lanes 2 and 3) and recombinant M1 (lane 4). Approximate protein sizes indicated by the molecular weight marker (kDa) are shown on the left.
(TIFF)

S2 Fig Immunogenicity of SV1 and M5 in rats. Antibody titres to (**A**) M5 and (**B**) SV1, (**C**) porcine cardiac myosin and (**D**) collagen type-1 following immunization of rats with SV1 or M5 in the presence of CFA or alum. No titres were observed against cardiac myosin or collagen type-1 in these assays.
(TIF)

S3 Fig Histological analysis of aortic, tricuspid and mitral valves following immunization of Lewis Rats with M5, SV1 or PBS admixed with alum. No signs in inflammation are visible in the myocardium (m), valves (v) or blood (b).
(TIF)

S1 Table Distribution of J14_i_ variants in GAS M-types. (PDF)
