# Supplementary figures and images for: Predicted Coverage and Immuno-Safety of a Recombinant C-Repeat Region Based Streptococcus pyogenes Vaccine Candidate
Source: PLoS One. 2016 Jun 16;11(6):e0156639. doi: 10.1371/journal.pone.0156639 (PMC4911098; doi:10.1371/journal.pone.0156639)

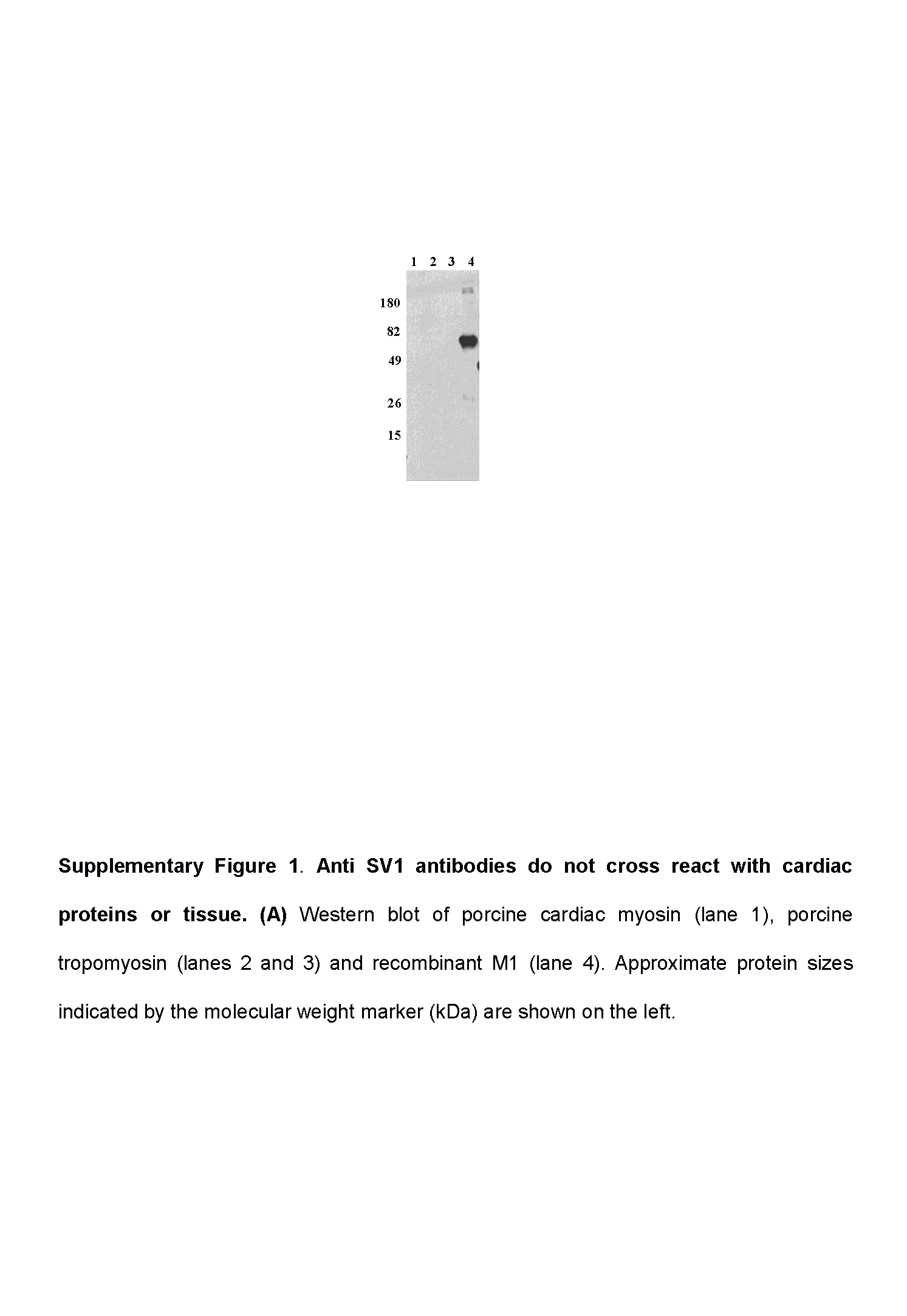

Supplement: S1 Fig — (A) Western blot of porcine cardiac myosin (lane 1), porcine tropomyosin (lanes 2 and 3) and recombinant M1 (lane 4). Approximate protein sizes indicated by the molecular weight marker (kDa) are shown on the left. (TIFF) [file pone.0156639.s001.tiff]

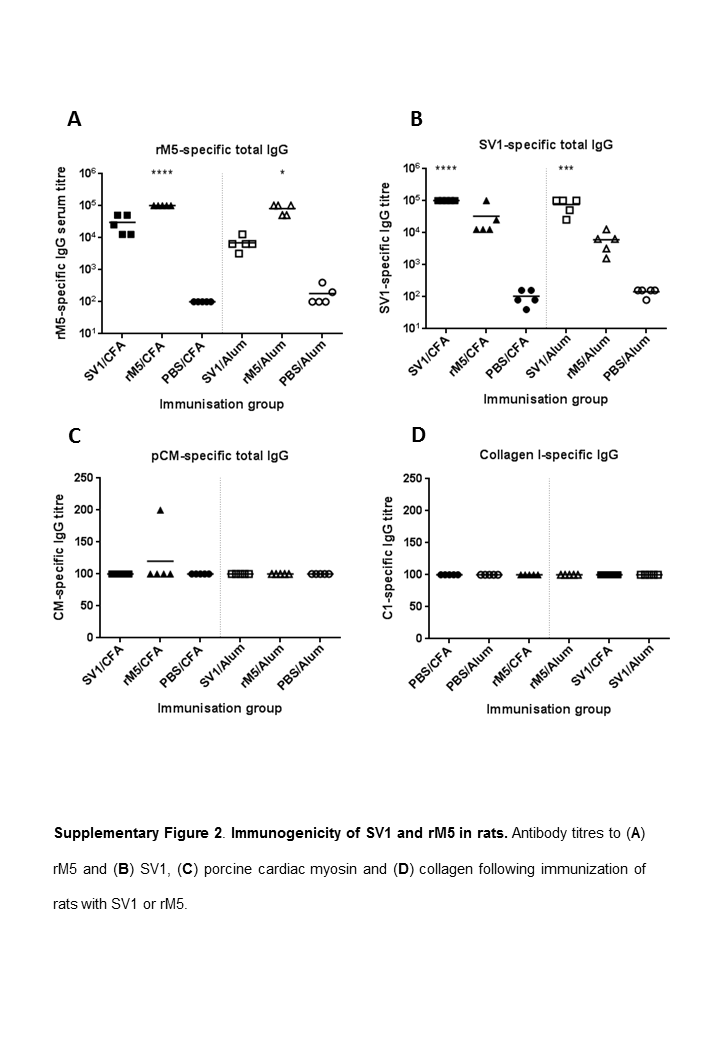

Supplement: S2 Fig — Antibody titres to (A) M5 and (B) SV1, (C) porcine cardiac myosin and (D) collagen type-1 following immunization of rats with SV1 or M5 in the presence of CFA or alum. No titres were observed against cardiac myosin or collagen type-1 in these assays. (TIF) [file pone.0156639.s002.tif]

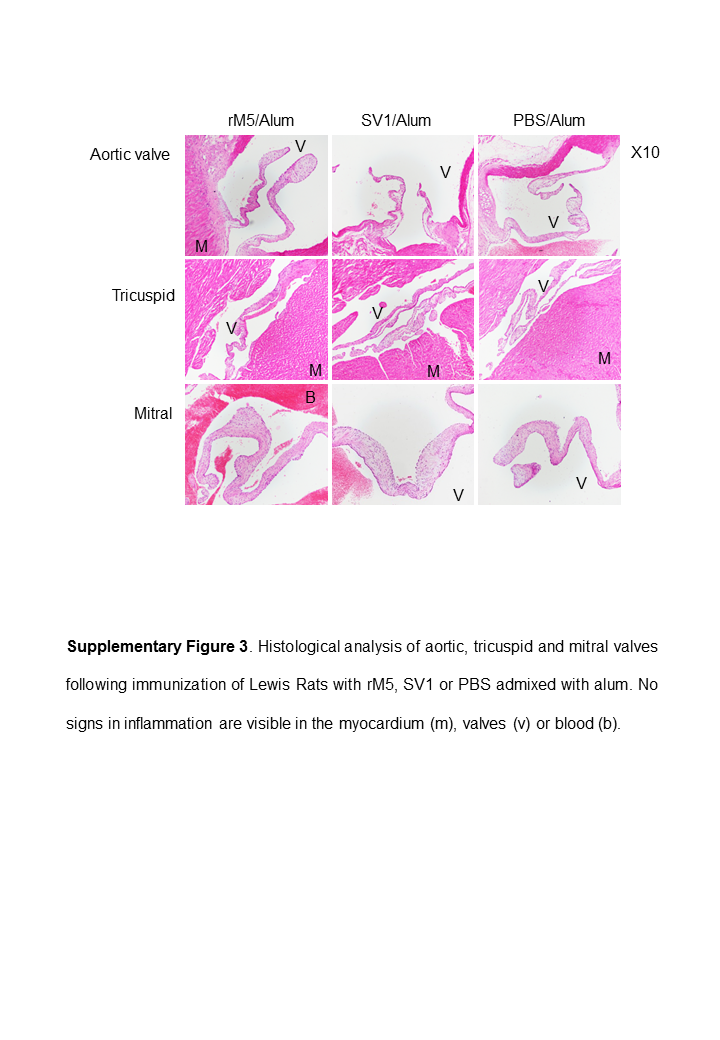

Supplement: S3 Fig — No signs in inflammation are visible in the myocardium (m), valves (v) or blood (b). (TIF) [file pone.0156639.s003.tif]
