# Supplementary material for: Predicted Coverage and Immuno-Safety of a Recombinant C-Repeat Region Based Streptococcus pyogenes Vaccine Candidate
Source: PLoS One. 2016 Jun 16;11(6):e0156639. doi: 10.1371/journal.pone.0156639 (PMC4911098; doi:10.1371/journal.pone.0156639)
Supplement: S1 Table — (PDF) [file pone.0156639.s004.pdf]

Supplementary Table 1. Distribution of $J14_i$-variants in different M-types[a].

emm-type	CN2	CN1	CRU1	CRU2	CRU3
1	J14.2	J14.2	J14.2	J14.2	J14.0
1-2	-	-	J14.2	J14.2	J14.0
1-4	-	-	J14.2	J14.2	J14.0
2	-	-	-	J14.29	J14.1
3	-	J14.84	J14.5	J14.2	J14.0
4	-	-	J14.1	J14.1	J14.1
5	-	-	J14.4	J14.2	J14.0
6	-	-	J14.4	J14.2	J14.0
8	-	-	J14.8	J14.1	J14.1
9	-	-	J14.29	J14.1	J14.1
11	-	-	J14.36	J14.1	J14.1
12	-	J14.84	J14.16	J14.2	J14.0
13	-	-	J14.8	J14.1	J14.1
14	-	-	J14.4	J14.4	J14.0
15	-	-	J14.29	J14.1	J14.1
18	-	-	J14.4	J14.2	J14.1
19	-	-	J14.4	J14.2	J14.0
22	-	-	J14.8	J14.1	J14.1
23	-	J14.4	J14.4	J14.2	J14.0
24	-	-	J14.4	J14.4	J14.0
25	-	-	J14.29	J14.1	J14.1
26	-	-	J14.4	J14.2	J14.0
27	-	-	-	J14.1	J14.1
28	-	-	J14.29	J14.41	J14.1
29	-	-	J14.4	J14.4	J14.0
30	-	-	J14.4	J14.4	J14.0
31	-	J14.84	J14.16	J14.2	J14.0
32	-	-	J14.2	J14.2	J14.0
33	-	-	J14.2	J14.2	J14.0
36	-	-	J14.4	J14.2	J14.0
39	-	-	J14.84	J14.4	J14.0
41	-	-	J14.2	J14.2	J14.0
42	-	-	J14.53	J14.1	J14.1
43	-	-	J14.2	J14.2	J14.0
44	-	-	J14.8	J14.1	J14.1
48	-	-	J14.36	J14.1	J14.1
49	-	-	J14.1	J14.1	J14.1
50	-	-	J14.8	J14.8	J14.1
52	-	-	J14.2	J14.2	J14.0
53	-	-	J14.4	J14.2	J14.0
54	-	-	J14.4	J14.2	J14.0

emm-type	CN2	CN1	CRU1	CRU2	CRU3
55	-	-	-	J14.2	J14.0
56	-	-	J14.2	J14.1	J14.1
57	J14.46	J14.46	J14.4	J14.2	J14.0
58	-	-	J14.29	J14.1	J14.1
59	-	-	J14.53	J14.1	J14.1
60	-	-	J14.12	J14.1	J14.1
63	-	-	J14.69	J14.1	J14.1
64	-	-	J14.2	J14.2	J14.0
65	-	-	J14.36	J14.1	J14.1
66	-	-	J14.1	J14.1	J14.1
67	-	-	J14.36	J14.36	J14.1
68	-	-	J14.8	J14.1	J14.1
70	-	-	J14.2	J14.1	J14.1
71	-	-	J14.4	J14.4	J14.0
73	-	-	J14.8	J14.1	J14.1
74	-	-	J14.4	J14.4	J14.0
75	-	-	J14.36	J14.1	J14.1
76	-	-	J14.29	J14.1	J14.1
77	-	-	J14.8	J14.1	J14.1
78	-	-	J14.12	J14.1	J14.1
79	-	-	J14.29	J14.1	J14.41
80	-	-	J14.2	J14.2	J14.0
81	-	-	J14.53	J14.1	J14.1
82	-	-	J14.72	J14.1	J14.1
83	-	J14.2	J14.2	J14.2	J14.0
84	-	-	J14.29	J14.1	J14.1
85	-	-	J14.53	J14.1	J14.1
86	-	-	J14.2	J14.2	J14.0
87	-	-	J14.8	J14.1	J14.1
88	-	-	J14.29	J14.1	J14.1
89	-	-	J14.1	J14.1	J14.1
90	-	-	J14.29	J14.1	J14.1
91	-	-	J14.2	J14.2	J14.0
92	-	-	J14.1	J14.1	J14.1
93	-	-	J14.2	J14.2	J14.0
94	-	J14.12	J14.12	J14.1	J14.1
95	-	-	-	J14.2	J14.1
96	-	-	J14.8	J14.1	J14.1
97	-	-	J14.2	J14.57	J14.0
98	-	-	J14.2	J14.2	J14.0
99	-	-	J14.36	j14.1	J14.1
100	-	-	-	J14.4	J14.0
101	-	-	J14.2	J14.2	J14.0

emm-type	CN2	CN1	CRU1	CRU2	CRU3
102	-	-	J14.29	J14.1	J14.1
103	-	-	J14.8	J14.1	J14.1
104	-	-	J14.1	J14.1	J14.1
105	-	-	-	J14.4	J14.0
106	-	-	J14.8	J14.1	J14.1
107	-	-	J14.29	J14.1	J14.41
108	-	-	J14.2	J14.2	J14.0
109	-	-	J14.8	J14.1	J14.1
110	-	-	J14.8	J14.1	J14.1
111	-	-	-	-	J14.0
112	-	-	J14.29	J14.1	J14.1
113	-	-	J14.8	J14.1	J14.1
114	-	-	J14.29	J14.1	J14.1
115	-	-	J14.4	J14.2	J14.0
116	-	-	J14.2	J14.2	J14.0
117	-	-	J14.8	J14.1	J14.1
118	-	-	J14.79	J14.1	J14.80
119	-	-	J14.2	J14.1	J14.1
120	-	-	J14.2	J14.1	J14.1
121	-	-	J14.1	J14.1	J14.1
122	-	-	-	J14.4	J14.2
123	-	J14.4	J14.2	J14.2	J14.0
124	-	-	J14.29	J14.1	J14.1
emm134	-	-	-	J14.12	J14.40
emm137	-	-	-	-	J14.12
emm174	-	-	-	J14.12	J14.40
emm205	-	-	-	J14.12	J14.40
emm211	-	-	-	J14.12	J14.40
st106M	-	-	J14.12	J14.12	J14.1
st11014	-	-	J14.12	J14.1	J14.1
st1207	-	-	J14.29	J14.1	J14.1
st1389	-	-	J14.8	J14.1	J14.1
st1692	-	-	-	J14.2	J14.0
st1731	-	-	J14.29	J14.1	J14.1
st1815	-	-	J14.12	J14.1	J14.1
st2037	-	-	J14.36	J14.1	J14.1
st212	-	-	J14.29	J14.1	J14.1
st213	-	-	J14.12	J14.1	J14.1
st2147	-	-	J14.55	J14.1	J14.1
st22	-	-	J14.2	J14.2	J14.0
st221	-	-	-	J14.2	J14.0
st2460	-	-	J14.29	J14.1	J14.1
st2861UK	-	-	J14.53	J14.1	J14.1

emm-type	CN2	CN1	CRU1	CRU2	CRU3
st2904	-	-	J14.58	J14.1	J14.1
st2911	-	-	J14.2	J14.2	J14.0
st2917	-	J14.53	J14.53	J14.1	J14.1
st2926	-	-	J14.2	J14.2	J14.1
st2940	-	-	J14.2	J14.2	J14.0
st369	-	-	J14.62	J14.1	J14.1
st3757	-	-	J14.2	J14.1	J14.1
st3765	-	-	-	J14.2	J14.0
st38	-	-	J14.2	J14.57	J14.0
st3850	-	-	J14.2	J14.2	J14.1
st4119	-	-	J14.4	J14.4	J14.0
st412	-	-	J14.2	J14.2	J14.0
st6030	-	-	J14.4	J14.2	J14.0
st6166B	-	-	J14.4	J14.2	J14.0
st62	-	-	J14.2	J14.57	J14.0
st6735	-	-	J14.8	J14.1	J14.1
st7323	-	-	J14.53	J14.1	J14.1
st7395	-	J14.2	J14.2	J14.2	J14.0
st7700	-	-	J14.4	J14.4	J14.0
st804	-	-	-	J14.4	J14.1
st809	-	-	J14.4	J14.2	J14.0
st818	-	-	J14.4	J14.2	J14.0
st854	-	-	J14.4	J14.2	J14.0
st9505	-	J14.8	J14.8	J14.8	J14.1
stck249	-	-	-	-	J14.0
stck401	-	-	-	J14.2	J14.0
std432	-	-	J14.2	J14.1	J14.1
stD631	-	-	J14.2	J14.2	J14.64
stD633	-	-	J14.2	J14.2	J14.0
stg4222	-	J14.62	J14.4	J14.60	J14.0
stg4545	-	-	J14.4	J14.2	J14.0
stg480	-	J14.46	J14.4	J14.60	J14.0
stg6	-	J14.4	J14.4	J14.60	J14.0
stg653	-	-	J14.4	J14.2	J14.59
stg866	-	-	J14.2	J14.2	J14.0
stil103	-	-	J14.2	J14.2	J14.0
stil62	-	-	-	J14.2	J14.0
stknb1	-	-	J14.35	J14.1	J14.1
stmd216	-	-	-	J14.2	J14.0
stn165	-	-	J14.62	J14.2	J14.0
stns1033	-	-	J14.2	J14.2	J14.0
stns292	-	-	J14.35	J14.1	J14.1
stns554	-	-	J14.58	J14.1	J14.1

emm-type	CN2	CN1	CRU1	CRU2	CRU3
stns90	-	-	J14.4	J14.4	J14.0
stpa57	-	-	-	J14.2	J14.0
sts104	-	J14.12	J14.29	J14.29	J14.0

[a]J14i variant present in SV1 are shown in colour
